# Effects of Long-Term Elevated CO_2_ on Soil Aggregate Structure and Microbial Communities in a *Deyeuxia angustifolia* Wetland of the Sanjiang Plain

**DOI:** 10.3390/microorganisms13122776

**Published:** 2025-12-05

**Authors:** Lanying Shi, Hongjie Cao, Rongtao Zhang, Haixiu Zhong, Yingnan Liu, Jifeng Wang, Donglai Zhang, Lin Li, Hongwei Ni

**Affiliations:** 1 School of Geographical Sciences, Harbin Normal University, Harbin 150025, China; 2 Department of Geography, College of History and Culture, Mudanjiang Normal University, Mudanjiang 157012, China; 3 Institution of Natural Resources and Ecology, Heilongjiang Academy of Sciences, Harbin 150040, China; hjcao781228@163.com (H.C.); zhangrongtao14@163.com (R.Z.); zhx971030@163.com (H.Z.); liuyingn234@163.com (Y.L.); wjfeng88@163.com (J.W.); 4 Heilongjiang Ecology Institute, Harbin 150081, China; slkyzdl@163.com (D.Z.); lilin_1002@163.com (L.L.); 5 Heilongjiang Academy of Forestry, Harbin 150081, China

**Keywords:** soil aggregates, aggregate stability, soil microorganisms, elevated atmospheric CO_2_

## Abstract

To investigate the effects of long-term elevated atmospheric CO_2_ (eCO_2_) on the distribution and stability of soil aggregates and microbial characteristics in wetland soils and to reveal the mechanisms by which eCO_2_ influences soil organic carbon (SOC) sequestration, a multi-temporal-scale eCO_2_ control experiment was conducted in the Sanjiang Plain wetland with treatments at ambient CO_2_ concentration (AC), 550 ppm, and 700 ppm CO_2_. Soil aggregate fractionation, phospholipid fatty acid (PLFA) analysis, and redundancy analysis (RDA) were used to analyze changes in aggregate size distribution, stability indices (MWD, GMD), microbial biomass, and community structure. The results showed that eCO_2_ significantly affected aggregate size distribution. Both short- and long-term exposure to low-concentration eCO_2_ reduced the proportion of large aggregates. Over time, the proportion of silt and clay particles increased, while microaggregates decreased. Although CO_2_ concentration did not directly affect MWD and GMD, long-term eCO_2_ significantly reduced soil aggregate stability. Microbial biomass and diversity were not sensitive to CO_2_ concentration but decreased significantly with prolonged exposure. In contrast, microbial community structure was significantly affected by both CO_2_ level and exposure duration. RDA indicated that, under short-term eCO_2_, aggregate fractions were positively correlated with microbial biomass, whereas, under medium- and long-term treatments, they were positively correlated with soil physicochemical properties. Macroaggregates were positively correlated with aggregate stability, while microaggregates and silt–clay fractions were negatively correlated—a relationship that strengthened with longer eCO_2_ exposure. Thus, long-term eCO_2_ altered soil aggregate structure and microbial communities, ultimately influencing SOC stability. These findings provide data and theoretical support for predicting soil carbon stability and ecosystem functioning in wetlands under climate change.

## 1. Introduction

Since the Industrial Revolution, human activities—including large-scale fossil fuel combustion and land use changes—have caused a nearly 50% increase in atmospheric carbon dioxide (CO_2_) concentration from 280 ppm in 1750 to approximately 416 ppm in 2022 [1]. According to the report of the Intergovernmental Panel on Climate Change (IPCC), the greenhouse gas emissions—primarily CO_2_—already caused global temperatures to rise by 0.8–1.2 °C above pre-industrial levels by 2017 [2]. Under current emission trends, global warming is projected to reach 1.5 °C between 2030 and 2052. Such changes are expected to intensify risks to natural and human systems and introduce new threats. Limiting temperature rise to 1.5 °C has thus become an urgent global priority. Addressing this challenge requires a dual approach: reducing greenhouse gas emissions—especially CO_2_—to directly curb atmospheric concentrations, and enhancing ecosystem carbon sinks to mitigate atmospheric CO_2_ levels and slow global warming.

Soil aggregates are fundamental units of soil structure [3,4]. In particular, macroaggregates (>0.25 mm) play a crucial role in protecting soil organic carbon (SOC) from microbial degradation, representing a key mechanism for SOC stabilization [5,6]. The stability of soil aggregates directly affects the soil carbon cycling process [7]. Elevated atmospheric CO_2_ (eCO_2_) significantly affects the distribution and stability of soil aggregates, though responses vary across ecosystems. For instance, in farmland ecosystems, long-term eCO_2_ exposure (9 years) has been shown to promote the breakdown of macroaggregates and the formation of microaggregates [8]. In contrast, studies on Mollisol indicated that eCO_2_ may enhance aggregate stability [9]. ECO_2_ has the potential to enhance the structural stability of Mollisol aggregates. These discrepancies may originate from the intrinsic soil properties: the decomposition process of organic matter in paddy soil is comparatively slow under flooded conditions, whereas Mollisol possesses a high organic matter and aggregation potential.

Soil microorganisms are essential to the formation and stabilization of soil aggregates. Through hyphal entanglement (e.g., by arbuscular mycorrhizal fungi), secretion of extracellular polymeric substances (e.g., polysaccharides and glycoproteins), and metabolic by-products such as organic acids, microbes facilitate the binding of soil particles into stable micro- and macroaggregates [10]. Conversely, aggregates provide physical protection for microorganisms, offering refuge from predation and desiccation, and creating niches enriched in organic matter and nutrients [11]. This reciprocal relationship—where microbes promote aggregation and aggregates influence microbial community composition—underpins critical soil processes including carbon sequestration, nutrient cycling, and overall ecosystem sustainability. A deeper understanding of these interactions is essential for predicting soil responses to global change.

ECO_2_ generally enhances microbial activity and aggregate stability by altering carbon dynamics in plant–soil systems [12]. On the one hand, high CO_2_ concentrations typically stimulate plant photosynthesis, augment the input of root exudates and litter, thereby offering a more abundant carbon source and energy for soil microorganisms. This response is particularly pronounced in C_3_ plants, which dominate temperate wetlands and exhibit photosynthetic enhancement of 20–50% under eCO_2_ due to reduced photorespiration [13,14]. The dominant species in this study, *Deyeuxia angustifolia*, is a C_3_ plant [15]; unlike C_4_ species (e.g., Spartina in coastal marshes wetland) that show minimal CO_2_ fertilization at current levels, C_3_ *D. Angustifolia* likely sustains elevated root exudation and biomass allocation under 550–700 ppm, driving microbial proliferation and aggregate formation in the anaerobic rhizosphere. This C_3_-specific CO_2_ sensitivity provides a strong mechanistic rationale for expecting robust plant–soil feedbacks in Sanjiang wetlands, distinguishing them from C_4_-dominated systems. This stimulates their activity and biomass (particularly that of mycorrhizal fungi associated with the carbon cycle) and may lead to changes in the microbial community structure [16]. On the other hand, the enhanced microbial activity and the growth of fungal mycelium generate more microbial by-products (such as glomalin-related soil protein, GRSP) and extracellular polymers. These substances, serving as crucial “glue” materials, can effectively bind soil particles together, facilitate the formation of large aggregates, and improve their stability [17]. Although previous research has reported that eCO_2_ promotes the formation of macroaggregates (particle size > 0.25 mm) in forest, farmland, and wetland ecosystems [18,19,20], other studies have indicated that eCO_2_ can reduce the proportion and stability of macroaggregates in wetlands [21,22]. This finding suggests that the response mechanism of the “soil aggregate-microorganism” system remains poorly understood. Therefore, it is imperative to investigate the specific patterns and mechanisms underlying the “carbon protection” function of this system in the wetland of the Sanjiang Plain.

The Sanjiang Plain Wetland is a critical ecological region in Northeast Asia, with significant roles in biodiversity conservation and carbon sequestration. However, rising atmospheric CO_2_ and other climate-change factors threaten this ecosystem, potentially leading to biodiversity loss, increased greenhouse gas emissions, and soil nutrient depletion. Although eCO_2_ effects on aggregate dynamics have been examined in agricultural [18,19] and forest [20] systems, wetlands of the Sanjiang Plain differ fundamentally in hydrology, redox regime, and organic-matter quality. Seasonal waterlogging (July–August) creates persistent anaerobic microsites that favor methanogenesis and Fe/Al-mediated organo-mineral complexation rather than the aerobic, tillage-driven turnover typical of croplands or the deep litter inputs of forests. These conditions decouple plant-C inputs from rapid microbial oxidation, potentially shifting long-term C protection from physical (aggregate occlusion) to chemical (mineral association) mechanisms—a transition rarely captured in short-term or non-wetland studies. The 14-year chronosequence (1-, 9-, and 14-year open-top chambers exposures) spanning short- to long-term eCO_2_ (550 and 700 ppm) therefore provides a rare temporal gradient to test whether aggregate-microbial feedbacks follow the linear stimulation observed in uplands or exhibit threshold responses under prolonged anaerobiosis and nutrient limitation. By integrating wet-sieving, PLFA profiling, and RDA across this gradient, we reveal stage-specific shifts from microbial-driven macroaggregation (short-term) to mineral-driven fine-fraction stabilization (long-term)-insights that refine wetland-specific C models and inform conservation under rising CO_2_.

This study investigated the effects of eCO_2_ on the structure, stability of soil aggregates, and the composition of the soil microbial community in a typical *D. angustifolia* wetland in the Sanjiang Plain. By using open-top chambers (OTCs) to simulate eCO_2_ conditions, this research tested two hypotheses: (1) eCO_2_ altered the structure and stability of soil aggregates, and (2) eCO_2_ modified the soil microbial community. The findings provide significant insight into potential impacts of future climate change on wetland soil and support the development of effective conservation strategies for the wetland.

## 2. Materials and Methods

### 2.1. Study Site and Experimental Setup

The research was conducted at the Sanjiang Plain Wetland Ecosystem Research Station of the Institute of Natural and Ecological Research, Heilongjiang Academy of Sciences (47°35′ N, 133°31′ E). The altitude of this research station ranges from 55 to 65 m above sea level. The average temperature is approximately 1.9 °C. The effective accumulated temperature above 10 °C lies between 2165 and 2624 °C. The lowest temperature in January reaches −20.4 °C, and the highest temperature in July is 21.6 °C. The mean annual precipitation is 585 mm, concentrated from July to September, and the mean annual evaporation is 1166 mm. *D. angustifolia* is the dominant species in this area. The soil is classified as swamp peat soil and meadow swamp soil, featuring seasonal waterlogging in July and August [23]. The experiment simulating atmospheric eCO_2_ was carried out using open-top chambers (OTCs). The device was built with an octagonal ring structure with a height of 1.8 m, covering a total plot area of ca 10.78 m^2^ covered with a polycarbonate plate (90% light transmittance). We supplied carbon dioxide to the chambers through a pipe with pinholes connected to industrial CO_2_ tanks outside the chambers. We adjusted the CO_2_ supply in accordance with wind speed and CO_2_ concentrations by taking constant measurements with an infrared gas analyzer. The 27 OTCs were established following a randomized complete block design across a homogeneous 1.2-ha plot. The site was pre-surveyed for micro-topography, soil texture, and initial SOC; three spatial blocks (separated by >15 m buffers) were delineated to capture subtle hydrological gradients. Each block contained one full set of the nine treatment combinations, yielding true replication (n = 3) and minimizing spatial pseudo-replication.

The chambers, installed over a 14-year period, were used to represent a chronosequence of exposure durations, long-term (LT, 14 years), medium-term (MT, 9 years), and short-term (ST, 1 year), and the details of experiment design are shown in Table S1. Each exposure group was subjected to three CO_2_ treatments: ambient CO_2_ (AC), 550 ppm (EC1), and 700 ppm (EC2). Microclimate was continuously monitored using infrared CO_2_ sensors (GMT222, Vaisala, Vantaa, Finland) and temperature–humidity probes (HMP155, Vaisala) logging at 30 min intervals. CO_2_ targets (550 ± 30 ppm; 700 ± 40 ppm) were maintained >95% of the time; interior temperatures were 0.8–1.3 °C above ambient, and relative humidity 2–4% higher. The treatments were as follows: (1) short-term AC treatment; (2) short-term EC1 treatment; (3) short-term EC2 treatment; (4) medium-term AC treatment; (5) medium-term EC1 treatment; (6) medium-term EC2 treatment; (7) long-term AC treatment; (8) long-term EC1 treatment; and (9) long-term EC2 treatment.

### 2.2. Soil Sample Collection

In mid-July 2023, soil samples were collected from the 0–20 cm depth in each OTC. Five random sampling points were selected per chamber, and the soils were homogenized into one composite sample per chamber. A total of 27 soil samples were obtained. Samples from five randomly selected sampling points within each OTC gas chamber were thoroughly blended. After removing visible debris, plant residues, and gravel, all blended samples were partitioned into two portions. One portion was placed within a sterile bag inside an insulated box equipped with ice packs and subsequently transported to the laboratory. The sterile bag was stored at 4 °C for the measurement of various indicators, including soil microbial biomass, and the relevant experiments were concluded within a week. The other portion of the sample was naturally air-dried for the assessment of soil properties and sieving of soil aggregates.

### 2.3. Soil Aggregate Fractionation and Stability Indices

To minimize slaking artifacts, air-dried samples were pre-wetted by capillary action: 100 g of field-moist equivalent soil (adjusted to ~10% gravimetric water content) was placed on tension tables at −0.3 kPa for 30 min until uniformly moistened. The same mixed soil samples were first moistened. Subsequently, distilled water was slowly added, and the samples were allowed to stand for 10 min before undergoing the wet-sieving operation in the aggregate analyzer. The sample was subjected for 15 min to an amplitude of 3 cm and a frequency of 30 times per minute. Aggregates of different particle sizes, particularly those >2 mm (large macroaggregates), 0.25–2 mm (small macroaggregates), 0.053–0.25 mm (microaggregates), and <0.053 mm (silt and clay particles), were separately collected in aluminum boxes. After the water-stable aggregates were dried at 105 °C for 12 h, the mass of each particle size fraction was calculated.

The mean weight diameter (MWD, mm) and geometric mean diameter (GMD, mm) were calculated using Equations (1) and (2) [5] as follows:

(1) $$MWD=\sum_{i=1}^{n}Wi\bar{di}$$

(2) $$GMD=\exp\left[\frac{\sum_{i=1}^{n}Wi\bar{di}}{\sum_{i=1}^{n}Wi}\right]$$

where $\bar{di}$ and Wi represent the average diameter (mm) and the weight percentage of water-stable aggregates, respectively, for each observed particle size.

### 2.4. Soil Sample Analyses

Total nitrogen (TN) and SOC contents were determined using an elemental analyzer (Model 2400; PerkinElmer, Shelton, CT, USA). Soil pH was measured using a pH meter (Mettler-Toledo, Columbus, OH, USA) at a soil-to-water ratio of 1:2.5 (*w*/*v*). Nitrate nitrogen [24] and ammonium nitrogen [25] contents were determined using colorimetry 48 h after extraction. The available nitrogen (AN) content was calculated as the sum of these two forms of nitrogen (N). Total soil P(TP) and available P (AP) contents were determined using the sodium hydroxide fusion-Mo-Sb anti-colorimetric method and 0.5 mol·L^−1^ sodium bicarbonate extraction-Mo-Sb anti-colorimetric method, respectively [26]. Dissolved organic C (DOC) was extracted using 0.5 mol·L^−1^ potassium sulfate at a soil-to-solution ratio of 1:4 (*w*/*v*) and measured with a total organic C (TOC) analyzer (Vario TOC, Elementar, Langenselbold, Germany). The permanganate-oxidizable organic carbon (PPOC) was measured using the method proposed by Logninow et al. [27], while the acid-hydrolyzable organic carbon (AHOC) was measured according to the method described by Rovira and Vallejo (2002) [28]. Soil microbial biomass C (MBC), microbial biomass N (MBN), and microbial biomass P contents were determined using the fumigation–extraction method [29].

### 2.5. Phospholipid Fatty Acid Analysis (PLFA Analysis)

We selected PLFA analysis because it provides quantitative estimates of viable microbial biomass and broad community composition, is cost-effective for large sample sets, and allows direct comparison with decades of prior CO_2_-enrichment studies that used the same method [19,20]. Although 16S/ITS sequencing offers higher taxonomic resolution, PLFA remains superior for biomass quantification and detecting physiologically relevant shifts in major groups under long-term treatments. The PLFAs were extracted and identified following the protocols [30,31]. Briefly, freeze-dried and sieved (2 mm) soil samples (2 g) were extracted twice with a single-phase chloroform–methanol–citrate buffer mixture (1:2:0.8 *v*/*v*/*v*, 0.15 mol∙L^−1^, pH 4.0). Approximately 2 g of the soil samples were subjected to two extractions using 22.8 mL of a single-phase mixture of chloroform–methanol–citrate buffer (1:2:0.8 *v*/*v*/*v*, 0.15 mol L^−1^, pH 4.0). Subsequently, the phospholipids were separated from neutral lipids and glycolipids using a silica gel column (Supelco, Bellefonte, PA, USA). Nonadecanoic acid methyl ester fatty acid (19:0) was added as an internal standard for the quantification of the phospholipid concentration prior to further analysis. Following the methylation of the phospholipids, the PLFA methyl esters were separated and identified via gas chromatography (GC; N6890, Agilent, Santa Clara, CA, USA) equipped with MIDI Sherlock microbial identification software (Version 4.5, MIDI, Newark, DE, USA). The 19:0 methyl ester was employed as an internal standard to guarantee accurate quantification. The following PLFAs were used as markers for specific microbial groups: i14:0, i15:0, a15:0, i16:0, i17:0, and a17:0 for Gram-positive bacteria; 16:1ω5c, 17:1ω8c, cy17:0, and cy19:0 for Gram-negative bacteria. Among these PLFAs, 18:1ω9c and 10Me16:0 PLFAs served as biomarkers for fungi, while 10Me17:0 and 10Me18:0 PLFAs functioned as biomarkers for actinomycetes. Non-specific (general) PLFAs were characterized by straight-chain acids, including 14:0, 15:0, 16:0, 17:0, and 18:0.

### 2.6. Statistical Analyses

We assessed the normality of variable distributions, applying a log_10_ transformation where necessary. One-way analysis of variance (One-way ANOVA) and Tukey’s test were utilized to examine the differences in aggregate mass ratio, aggregate stability, soil properties, microbial mass, and microbial diversity under varying eCO_2_ concentrations and different time treatments. Pearson correlation analysis was applied to ascertain the correlation between the aggregate mass ratio and aggregate stability indicators. Redundancy analysis (RDA) was utilized to discern the correlations among soil physical and chemical properties, microbial properties, and the mass ratios of aggregates with varying particle sizes. The analysis was executed using the Vegan package and the rdacca. pH package, and the plots were generated using the ggplot2 package. The RDA and plot generation were conducted using R v.4.3.0 software (https://www.r-project.org/).

## 3. Results

### 3.1. Particle Size Distribution of Soil Aggregates

**Figure 1:**
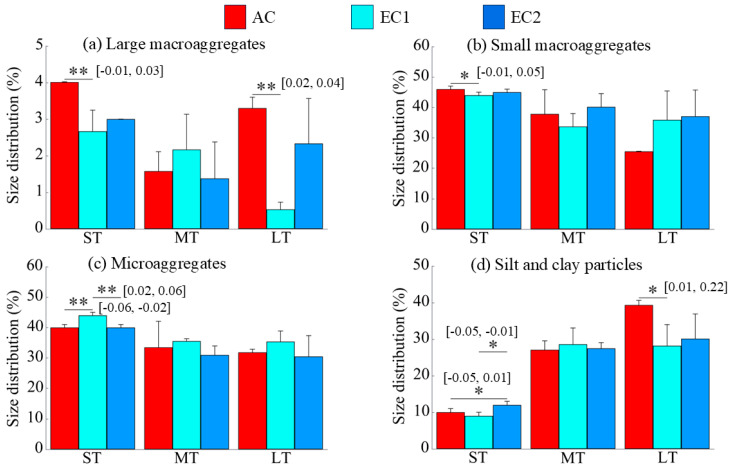
Changes in particle size distribution under eCO_2_. ST, MT, and LT denote short-, medium-, and long-term treatments, respectively. The “*” and “**” indicate significance levels with *p* < 0.05 and *p* < 0.01, respectively. The values in brackets are the 95% confidence intervals.

The effects of eCO_2_ on the size distribution of soil aggregates varied across treatments (Figure 1). Compared to ambient CO_2_ (AC), both ST and LT exposure to low-concentration eCO_2_(EC1) significantly reduced the proportion of large macroaggregates (>2 mm) (Figure 1a, *p* < 0.01). Short-term EC1 also significantly decreased the proportion of small macroaggregates (Figure 1b, *p* < 0.05), while significantly increasing the proportion of microaggregates (0.053–0.25 mm) (*p* < 0.01; Figure 1c). Changes in the silt and clay fraction (<0.053 mm) were divergent. Specifically, the proportion of silt and clay particles increased significantly under short-term high-concentration eCO_2_(EC2) but exhibited a significant downward trend under long-term low-concentration eCO_2_ (Figure 1d, *p* < 0.05).

**Figure 2:**
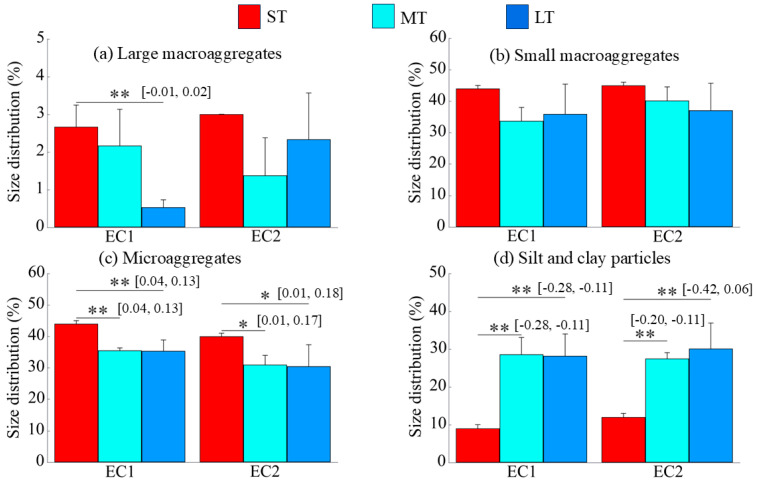
Changes in particle size distribution over the duration of eCO_2_. EC1 and EC2 denote the treatments with eCO_2_ at 550 ppm and 700 ppm, respectively. The “*” and “**” indicate significance levels with *p* < 0.05 and *p* < 0.01, respectively. The values in brackets are the 95% confidence intervals.

The duration of eCO_2_ exposure exerted a more consistent influence on different particle sizes of soil aggregates. Under the EC1 and EC2, prolonged exposure led to a significant increase in the silt and clay fraction (*p* < 0.01; Figure 2d) and a significant decrease in microaggregates (*p* < 0.05; Figure 2c), while the proportions of large and small macroaggregates generally declined (Figure 2a,b).

### 3.2. Soil Aggregate Stability

**Figure 3:**
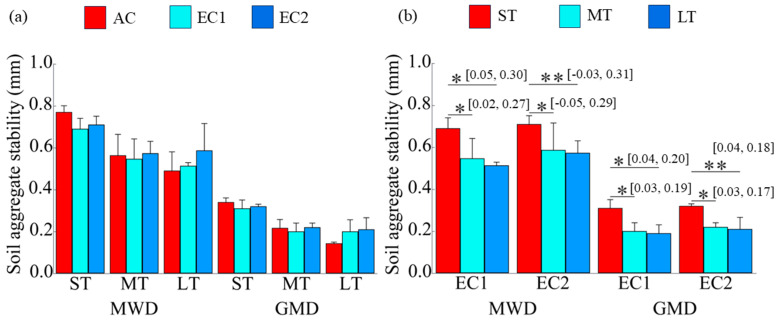
Changes in soil aggregate stability under eCO_2_ and the duration of eCO_2_. MWD denotes mean weight diameter, and GMD denotes geometric mean diameter. The “*” and “**” indicate significance levels with *p* < 0.05 and *p* < 0.01, respectively. The values in brackets are the 95% confidence intervals.

Compared to the AC treatment, eCO_2_ did not induce statistically significant changes in mean weight diameter (MWD) or geometric mean diameter (GMD)—key indicators of aggregate stability—across short-, medium-, or long-term exposures (*p* > 0.05; Figure 3a). However, the duration of eCO_2_ exposure significantly influenced aggregate stability, as evidenced by a gradual decline in both MWD and GMD with increasing exposure time (*p* < 0.05; Figure 3b).

### 3.3. Soil Microbial PLFAs, Composition, and Diversity

Phospholipid fatty acid (PLFA) analysis revealed that eCO_2_ concentration alone did not significantly alter total microbial biomass, bacterial biomass, fungal biomass, or the fungal-to-bacterial ratio (F/B) within any exposure period (*p* > 0.05; Table 1). In contrast, exposure duration had a pronounced effect. Total PLFAs, bacterial PLFAs, fungal PLFAs, and the F/B ratio all increased significantly over time under eCO_2_ (*p* < 0.05; Table 2). This temporal increase was particularly evident under EC1. Under EC2, microbial biomass parameters showed no significant difference between short-term (ST) and medium-term (MT) exposures but were significantly higher in the long-term (LT) treatment (*p* < 0.05; Table 2).

Analysis of alpha diversity indicated that the Shannon diversity index was not significantly affected by CO_2_ concentration (*p* > 0.05). However, the ACE index, a measure of microbial richness, decreased significantly under long-term eCO_2_ exposure (*p* < 0.05; Table 1). The response of the ACE index to exposure duration varied with concentration: it increased over time under EC1 but peaked at medium-term and was lowest in the long-term under EC2 (*p* < 0.05; Table 2). Permutational multivariate analysis of variance (PERMANOVA) confirmed that both CO_2_ concentration and exposure duration significantly influenced overall microbial community structure (Figure S1, *p* < 0.05).

### 3.4. Influencing Factors of Soil Aggregates

**Figure 4:**
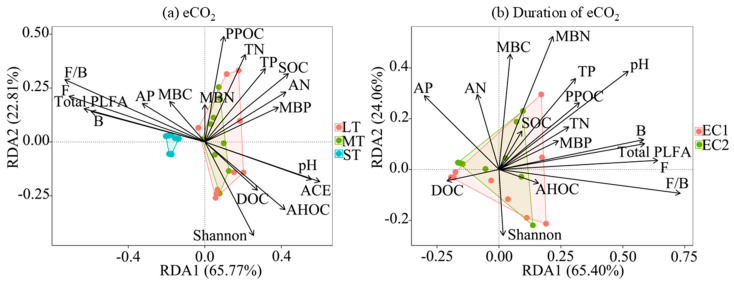
Influencing factors of soil aggregate fractions under eCO_2_ and duration of eCO_2_. RDA axes 1 and 2 accounted for 65.77% and 22.81% of the variation, respectively, under eCO_2_ at different times (**a**) and for 65.40% and 24.06%, respectively, with duration under different CO_2_ levels (**b**). The arrows represent explanatory variables; the length of an environmental arrow represents the strength of its influence on the soil aggregate composition. Furthermore, the cosine of the angle between any two arrows approximates their correlation, with acute, obtuse, and right angles indicating positive, negative, and no correlation, respectively. The arrows represent the explanatory variables, whereas the colored dots denote the distribution of data points for the soil aggregate fractions. The length of an arrow indicates the strength of its influence on the composition, and the angle between arrows reflects their correlation: acute, obtuse, and right angles indicate positive, negative, and no correlation, respectively. pH, soil acidity or alkalinity; SOC, soil organic carbon; TN, total soil nitrogen; TP, total phosphorus; DOC, dissolved organic carbon; PPOC, permanganate-oxidizable organic carbon; AHOC, acid-hydrolyzable organic carbon; AP, available phosphorus; AN, available nitrogen; MBC, microbial biomass carbon; MBN, microbial biomass nitrogen; MBP, microbial biomass phosphorus; Shannon, alpha diversity index; and ACE, microbial richness index.

RDA revealed significant associations between aggregate fractions and soil microbial and physicochemical properties (Figure 4). Under short-term eCO_2_ (ST), a positive correlation was observed between aggregate distribution and microbial biomass (total, bacterial, and fungal PLFAs), along with the F/B ratio. In medium-term (MT) and long-term (LT) treatments, aggregate fractions were strongly associated with soil physicochemical properties, including permanganate-oxidizable carbon (PPOC), total nitrogen (TN), total phosphorus (TP), pH, SOC, available nitrogen (AN), microbial biomass phosphorus (MBP), dissolved organic carbon (DOC), acid-hydrolyzable organic carbon (AHOC), and microbial alpha diversity indices (Shannon and ACE).

Regarding the influence of eCO_2_ treatment time, the association patterns between soil aggregate fractions and microbial and physicochemical properties under low-concentration (EC1) and high-concentration (EC2) eCO_2_ conditions were fundamentally consistent. Specifically, they were significantly correlated with indicators such as total PLFAs, bacterial PLFAs, fungal PLFAs, F/B, pH, MBN, MBC, and AP (Figure 4).

### 3.5. The Relationship Between Aggregate Components and Stability

Across all eCO_2_ exposure periods, large and small macroaggregates were consistently positively correlated with aggregate stability (i.e., MWD and GMD), whereas microaggregates and the silt–clay fraction were negatively correlated (Table 3). As exposure duration increased under both EC1 and EC2, the positive correlation of macroaggregates and the negative correlation of the silt–clay fraction with stability were reinforced.

## 4. Discussion

### 4.1. Changes in Soil Aggregate Distribution

In accordance with our first hypothesis, both short- and long-term eCO_2_ significantly reduced the proportion of large macroaggregates (>2 mm), whereas prolonged exposure under both 550 and 700 ppm markedly increased the silt+clay fraction (<0.053 mm) at the expense of microaggregates. This pattern suggests initial disruption of macroaggregates followed by progressive fragmentation over time. Enhanced root exudation under eCO_2_ likely stimulated microbial turnover of binding agents, accelerating the breakdown of large aggregates in the early stage, while cumulative carbon inputs and changing redox conditions favored the accumulation of fine particles in the long term. This process was markedly intensified under high-concentration eCO_2_ (EC2), which led to a 23.44% decrease in silt–clay content and a 45.20% increase in small macroaggregates—likely driven by enhanced root secretion and microbial activity that promoted the production of organic cementing agents [32]. Second, soil microorganisms, particularly fungi, were positively linked to aggregate evolution. Fungal PLFAs showed a significant positive correlation with the proportion of small macroaggregates (*p* < 0.05), supporting the known role of hyphal entanglement and glomalin secretion in aggregate formation [33]. These trends are consistent with molecular studies across ecosystems. For example, 16S rRNA sequencing in wetland systems revealed eCO_2_-induced enrichment of Actinobacteria and Acidobacteria and a decline in Proteobacteria, linked to increased rhizodeposition [34]. ITS profiling in temperate bogs showed increased Basidiomycota under eCO_2_, correlating with macroaggregate stability [35]. Metagenomic evidence further indicated upregulation of fungal polysaccharide synthesis genes under eCO_2_, reinforcing the role of fungal activity in cementing aggregates [36]. While these molecular insights align with and extend our PLFA-based trends, they also highlight a key limitation of our approach: the inability to resolve taxonomic or functional gene-level mechanisms. Future studies should integrate metagenomic and transcriptomic tools to directly link microbial functional traits with aggregate dynamics under eCO_2_. Such work would help clarify the causal—not just correlative—pathways through which eCO_2_ reshapes soil structure and carbon cycling.

### 4.2. Changes in Soil Aggregate Stability

Contrary to some upland studies, prolonged eCO_2_ did not increase aggregate stability in this wetland system (Figure 3a); instead, both MWD and GMD declined significantly with exposure duration (Figure 3b), even under ambient CO_2_. This consistent temporal decrease primarily reflects natural structural aging caused by repeated freeze–thaw cycles, root turnover, and redox fluctuations typical of wetland soils. The lack of a significant CO_2_ × duration interaction indicates that eCO_2_ accelerates, rather than initiates, this aging process. The observed reduction in stability aligns with the decreasing proportion of macroaggregates and increasing silt + clay fraction under long-term eCO_2_ (Table 3). Aggregate stability is closely tied to the abundance of macroaggregates (>0.25 mm), which provide physical protection for SOC [37,38,39,40,41,42]. In contrast to terrestrial ecosystems where eCO_2_ often strengthens microbe–mineral interactions and promotes stable aggregates [18,19], prolonged eCO_2_ here intensified microbial metabolism and nutrient limitation under anaerobic conditions, leading to aggregate breakdown and the mineralization of previously protected carbon.

Elevated CO_2_ also significantly influences the chemical composition and spatial distribution of soil organic carbon (SOC). Under long-term eCO_2_, SOC within microaggregates becomes structurally simpler and less diverse [43]. Synchrotron-based infrared microspectroscopy reveals that organic components in microaggregates distribute more homogeneously and interact strongly with clay mineral hydroxyl groups (O–H), thereby enhancing SOC stability [44]. However, in coarse-textured soils such as calcareous types, mineral–carbon associations weaken, potentially compromising physical protection mechanisms [45]. Moreover, eCO_2_ can substantially reduce both particulate organic carbon (POC) and mineral-associated organic carbon (MAOC), particularly in deeper soil layers (20–40 cm) [46]. Pyrolysis–GC/MS further indicates a decline in the diversity of organic compounds within MAOC under eCO_2_, reducing its chemical stability and influencing soil carbon persistence [47,48].

These duration-dependent, nonlinear effects reveal a staged aggregate–microorganism feedback: short-term stimulation of microbial binding agents is overtaken by longer-term nutrient limitation, microbial succession, and enhanced decomposition under fluctuating redox conditions. The rising silt + clay fraction indicates a shift from physical occlusion within macroaggregates to stronger chemical protection via organo-mineral complexes with reduced Fe/Al (hydr)oxides—a dominant stabilization pathway in wetlands. Thus, although wetland carbon inputs may increase under rising CO_2_, sequestration quality and long-term stability could decline unless mineral-association mechanisms are explicitly incorporated into future models.

### 4.3. Changes in Soil Microbial Community Structure

PLFA analysis revealed that eCO_2_ concentration alone exerted minimal influence on microbial biomass or richness within any exposure period. Instead, microbial biomass (total, bacterial, and fungal PLFAs) and the fungal–bacterial ratio increased significantly from short- to medium/long-term exposures, reflecting cumulative plant-derived carbon inputs from the dominant C_3_ vegetation (Table 1 and Table 2). In contrast, microbial richness (ACE index) and overall community structure responded strongly to the interaction of CO_2_ level and duration: long-term high-concentration eCO_2_ (700 ppm) caused the most pronounced decline in richness and the largest compositional shifts (*p* < 0.05). These changes were driven primarily by indirect effects rather than direct CO_2_ toxicity. Progressive soil acidification (Table S1) and depletion of total nitrogen and phosphorus created conditions favoring acid-tolerant, oligotrophic taxa such as Actinobacteria while suppressing more sensitive groups [49,50,51,52,53]. This is consistent with global CO_2_-enrichment experiments showing that sustained plant uptake without fertilization leads to progressive nutrient limitation, reduced diversity, and a shift toward slower-cycling microbial communities [53,54]. Although short-term eCO_2_ stimulated microbial biomass through enhanced rhizodeposition [55], prolonged exposure transitioned the regulatory mechanism from direct carbon stimulation to indirect control via altered soil chemistry and stoichiometry. The resulting community restructuring—particularly the enrichment of taxa capable of degrading recalcitrant carbon—may accelerate SOC mineralization under long-term high eCO_2_ [56], potentially weakening the carbon sink strength of unmanaged wetlands despite initial productivity gains.

Regarding the time effect, this study demonstrated that the duration of continuous exposure to eCO_2_ was a crucial environmental factor that affected the structure of soil microbial communities. This suggests that, as the duration of eCO_2_ exposure lengthened, the influencing mechanism of eCO_2_ gradually changes from directly stimulating biomass in the initial stage to indirectly regulating the structure of the microbial community by modifying the chemical properties of soil organic matter and nutrient cycling. A recent meta-analysis confirmed that eCO_2_ enhances the activity of carbon-degrading enzymes (e.g., cellulase, ligninase), correlating with increased SOC—highlighting time-dependent functional microbial responses [57], but enzyme activity or gene expression was not assessed here. Future work should use qPCR or metatranscriptomics to test whether PLFA increases reflect elevated C-degradation capacity.

While PLFA biomarkers effectively track shifts in major microbial groups (e.g., Gram-positive/negative bacteria, fungi, actinomycetes), they offer limited taxonomic resolution at the genus or species level and do not reflect functional gene abundance, expression, or enzyme activity [58]. For example, the observed increase in fungal PLFAs under long-term eCO_2_ is consistent with enhanced hyphal biomass but does not confirm specific roles in glomalin production or C decomposition. Similarly, changes in bacterial PLFA ratios may indicate community restructuring but cannot identify key taxa (e.g., Streptomyces or methanotrophs) driving aggregate dynamics. Future studies integrating high-throughput sequencing (16S rRNA, ITS) and metagenomics/transcriptomics are recommended to resolve functional microbial responses to eCO_2_ in this wetland system.

A key limitation of this chronosequence design is the potential confounding of exposure duration with inter-annual climate variability and initial site conditions. The ST, MT, and LT (OTCs) were established in different years across a homogeneous 1.2-ha plot, but subtle differences in weather (e.g., precipitation, temperature extremes) and pre-installation soil/vegetation states cannot be fully excluded despite randomized block design and pre-surveys. For instance, the LT cohort experienced 14 growing seasons, including extreme events (e.g., 2013 flood), while ST chambers saw only one. Such variability may contribute to observed microbial richness decline or aggregate shifts independently of eCO_2_ duration. While microclimate monitoring showed consistent OTC warming (+0.8–1.3 °C) and CO_2_ control (>95% target fidelity; Table S2), legacy effects remain unquantified. Thus, duration-related patterns should be interpreted as associational trends within a realistic field chronosequence, not strict causal outcomes of exposure time. True replication across multiple staggered sites would be needed to isolate duration effects—a logistical challenge for decadal eCO_2_ studies. Future work should pair chronosequences with continuous flux towers and isotopic (^13^C) labeling to disentangle climate, legacy, and eCO_2_ signals.

## 5. Conclusions

Prolonged elevated CO_2_ induces a nonlinear, stage-specific restructuring of soil aggregates and microbial communities in a temperate freshwater wetland. Short-term stimulation gives way to long-term aggregate fragmentation and a shift toward mineral-mediated SOC stabilization. These findings refine predictions of wetland carbon persistence under future atmospheric CO_2_ concentrations and highlight the need to incorporate duration-dependent microbial and mineral protection mechanisms into Earth system models.

## Figures and Tables

**Table 1:** PLFA concentrations (nmol g^−1^) and microbial community composition under eCO_2_.

Variables	Treatment								
	ST-AC-	ST-EC1	ST-EC2	MT-AC	MT-EC1	MT-EC2	LT-AC	LT-EC1	LT-EC2
Total PLFAs	83.16 ± 8.84 a	78.26 ± 4.58 a	89.27 ± 4.54 a	115.34 ± 9.19 a	136.06 ± 8.51 a	121.18 ± 10.92 a	125.16 ± 6.99 a	131.52 ± 4.26 a	149.88 ± 23.85 a
Bacterial PLFAs	33.42 ± 3.67 a	31.16 ± 1.73 a	35.27 ± 1.60 a	44.89 ± 3.31 a	52.86 ± 3.12 a	47.11 ± 4.23 a	47.28 ± 2.32 a	50.00 ± 1.02 a	56.73 ± 8.72 a
Fungal PLFAs	4.66 ± 0.46 a	5.04 ± 0.46 a	6.05 ± 0.51 a	9.90 ± 1.16 a	12.04 ± 1.25 a	10.87 ± 1.12 a	12.78 ± 1.29 a	12.53 ± 1.32 a	14.35 ± 2.65 a
F/B	0.14 ± 0.02 a	0.16 ± 0.01 a	0.17 ± 0.01 a	0.22 ± 0.01 a	0.23 ± 0.02 a	0.23 ± 0.01 a	0.27 ± 0.02 a	0.25 ± 0.02 a	0.25 ± 0.01 a
Alpha diversity									
Shannon	1.71 ± 0.02 a	1.72 ± 0.01 a	1.72 ± 0.01 a	1.72 ± 0.01 a	1.71 ± 0.01 a	1.72 ± 0.01 a	1.73 ± 0.01 a	1.72 ± 0.01 a	1.72 ± 0.01 a
ACE	10.34 ± 0.20 bc	10.26 ± 0.11 bc	10.38 ± 0.08 bc	10.92 ± 0.56 ab	11.27 ± 0.51 a	11.19 ± 0.86 a	11.40 ± 0.17 a	11.46 ± 0.10 a	10.03 ± 0.58 c

Within the same time period (short-term [ST], medium-term [MT], long-term [LT]), the presence of different lowercase letters corresponding to different concentrations signifies significant differences (*p* < 0.05), whereas the same lowercase letters indicate non-significant differences (*p* > 0.05).

**Table 2:** PLFA concentrations (nmol g^−1^) and microbial community composition under duration of eCO_2_.

Variables	Treatment					
	EC1-ST	EC1-MT	EC1-LT	EC2-ST	EC2-MT	EC2-LT
Total PLFAs	78.26 ± 4.58 a	136.06 ± 8.51 b	131.52 ± 4.26 b	89.27 ± 4.54 a	121.18 ± 10.92 ab	149.88 ± 23.85 b
Bacterial PLFAs	31.16 ± 1.73 a	52.86 ± 3.12 b	50.00 ± 1.02 b	35.27 ± 1.60 a	47.11 ± 4.23 ab	56.73 ± 8.72 b
Fungal PLFAs	5.04 ± 0.46 a	12.04 ± 1.25 b	12.53 ± 1.32 b	6.05 ± 0.51 a	10.87 ± 1.12 ab	14.35 ± 2.65 b
F/B	0.16 ± 0.01 a	0.23 ± 0.02 b	0.25 ± 0.02 b	0.17 ± 0.01 a	0.23 ± 0.01 b	0.25 ± 0.01 b
Alpha diversity						
Shannon	1.72 ± 0.01 a	1.71 ± 0.01 a	1.72 ± 0.01 a	1.72 ± 0.01 a	1.72 ± 0.01 a	1.72 ± 0.01 a
ACE	10.26 ± 0.11 c	11.27 ± 0.51 a	11.46 ± 0.10 a	10.38 ± 0.08 bc	11.19 ± 0.86 ab	10.03 ± 0.58 c

At the same concentration (550 ppm CO_2_ concentration [EC1], 700 ppm CO_2_ concentration [EC2]), different lowercase letters corresponding to different time points indicate significant differences (*p* < 0.05), while the same lowercase letters indicate non-significant differences (*p* > 0.05).

**Table 3:** Correlation between aggregate stability indices and the proportion of aggregates.

Treatments		GMD (mm)	>2 mm	0.25–2 mm	0.053–0.25 mm	<0.053 mm
ST	MWD	0.76 *	0.74 *	0.97 **	−0.18	−0.22
	GMD		0.60	0.73 *	−0.13	−0.12
MT	MWD	0.94 *	0.76 *	0.85 **	−0.74 *	−0.63
	GMD		0.54	0.94 **	−0.72 *	−0.75 *
LT	MWD	0.75 *	0.39	0.75 *	−0.86 **	−0.44
	GMD		0.30	0.99 **	−0.38	−0.92 **
EC1	MWD	0.91**	0.83 **	0.72 *	0.72 *	−0.87 **
	GMD		0.63	0.92 **	0.60	−0.92 **
EC2	MWD	0.85 **	0.77 *	0.86 **	0.15	−0.71 *
	GMD		0.53	0.86 **	0.50	−0.96 **

ST, MT, and LT denote short-, medium-, and long-term treatments, respectively; EC1 and EC2 denote the treatments with eCO_2_ at 550 ppm and 700 ppm, respectively; MWD denotes mean weight diameter, and GMD denotes geometric mean diameter. The “*” and “**” indicate significance levels with *p* < 0.05 and *p* < 0.01, respectively.

## Data Availability

The original contributions presented in this study are included in the article/Supplementary Material. Further inquiries can be directed to the corresponding authors.

## Supplementary Materials

The following supporting information can be downloaded at https://www.mdpi.com/article/10.3390/microorganisms13122776/s1, Table S1. Experimental design. Table S2. Characteristics of basic environmental factors of soil under eCO_2_ conditions. Table S3. Characteristics of basic environmental factors of soil under the duration of eCO_2_ conditions. Table S4. Influencing factors of soil aggregate fractions under eCO_2_ and duration of eCO_2_. Figure S1. Changes in soil microbial community structure. ST, MT, and LT denote short-term, medium-term, and long-term treatments respectively; AC, EC1, and EC2 denote treatments under ambient CO_2_ concentration, 550 ppm CO_2_ concentration, and 700 ppm CO_2_ concentration respectively. Figure S2. Changes in particle size structure under eCO_2_ and the duration of eCO_2._ ST, MT, and LT denote short-term, medium-term, and long-term treatments respectively; AC, EC1, and EC2 denote treatments under ambient CO_2_ concentration, 550 ppm CO_2_ concentration, and 700 ppm CO_2_ concentration respectively.
