# Supplementary material for: Effects of Long-Term Elevated CO2 on Soil Aggregate Structure and Microbial Communities in a Deyeuxia angustifolia Wetland of the Sanjiang Plain
Source: Microorganisms. 2025 Dec 5;13(12):2776. doi: 10.3390/microorganisms13122776 (PMC12735876; doi:10.3390/microorganisms13122776)
Supplement: Supplementary file 1 [file microorganisms-13-02776-s001.zip › microorganisms-3918204-supplementary.pdf]

Effects of elevated CO_2 on soil aggregate structure and microbial communities in a *Deyeuxia angustifolia* wetland of the Sanjiang Plain

Lanying Shi [1,3,*,†], Hongjie Cao [4,†], Rongtao Zhang[4], Haixiu Zhong [4], Yingnan Liu [4], Jifeng Wang [4], Donglai Zhang [5], Lin Li [5], Hongwei Ni [2,*]

[1] School of Geographical Sciences, Harbin Normal University, Harbin 150025, China; swxsly@126.com (L. S.)
[2] Heilongjiang Academy of Forestry, Harbin 150081, China
[3] Department of Geography, College of History and Culture, Mudanjiang Normal University, Mudanjiang 157012, China
[4] Institution of Natural Resources and Ecology, Heilongjiang Academy of Sciences, Harbin 150040, China
[5] Heilongjiang Ecology Institute, Harbin 150081, China
* Correspondence: nihongwei2000@163.com (H.N.)
† These authors contributed equally to this work.

Table S1 Experimental design

	Ambient CO_2 (AC)	550 ppm (EC1)	700 ppm (EC2)
Short-term (ST)	(1) short-term AC treatment	(2) short-term EC1 treatment	(3) short-term EC2 treatment
medium-term (MT)	(4) medium-term AC treatment	(5) medium-term EC1 treatment	(6) medium-term EC2 treatment
long-term (LT)	(7) long-term AC treatment	(8) Long-term EC1 treatment	(9) Long-term EC2 treatment

Physical and chemical properties of soil

Significant changes in soil physicochemical properties were observed under elevated CO_2 (eCO_2) conditions (Table S2, $P < 0.05$). Under the ST condition, low - concentration eCO_2 (EC1) significantly reduced microbial biomass carbon (MBC) compared to the ambient CO_2 (AC) conditions, whereas high-concentration eCO_2 (EC2) showed no significant effect.

Under long-term exposure (LT), EC2 resulted in a significant decrease in soil pH, and EC1 led to a significant reduction in microbial biomass nitrogen (MBN) (Table S2, $P < 0.05$). Under medium-term exposure (MT), EC2 significantly decreased the contents of AN and AP (Table S2, $P < 0.05$) compared to AC, while EC1 induced no notable changes. Under long-term exposure (LT), EC2 resulted in a significant decrease in soil pH, and EC1 led to a significant reduction in in MBN (Table S2, $P < 0.05$).

Prolonged exposure to eCO_2 further modulated these effects. Compared to ST, LT under both EC1 and EC2 significantly reduced soil pH (Table S3, $P < 0.05$). Under EC1, LT also significantly

decreased AP content compared to ST (Table S3, $P < 0.05$). Under EC2, MT significantly reduced AN and AP contents compared to ST (Table S3, $P < 0.05$).

Table S2 Characteristics of basic environmental factors of soil under eCO_2 conditions

Soil physio-chemical properties	ST			MT			LT		
	AC-	EC1	EC2	AC	EC1	EC2	AC	EC1	EC2
pH	5.27±0.08a	5.31±0.03a	5.25±0.04a	5.32±0.09a	5.38±0.05a	5.43±0.10a	6.11±0.20a	5.76±0.12a	5.62±0.06b
SOC (g kg^{-1})	25.69±0.20a	27.06±0.03a	30.98±3.17a	32.46±0.21a	31.51±1.69 a	32.86±3.56a	31.90±2.15a	30.17±1.84a	38.07±2.84a
TN (g kg^{-1})	3.70±1.18a	2.53±0.43a	2.85±0.43a	3.51±0.52a	4.07±0.37a	3.65±0.36a	3.16±0.53a	3.78±0.56a	4.30±1.00a
TP (g kg^{-1})	0.58±0.09a	0.46±0.02a	0.55±0.08a	0.61±0.06a	0.66±0.04a	0.66±0.04a	0.60±0.03a	0.65±0.06a	0.73±0.15a
DOC (g kg^{-1})	0.11±0.02a	0.12±0.01a	0.11±0.001a	0.16±0.03a	0.18±0.02a	0.17±0.03a	0.13±0.01a	0.13±0.02a	0.11±0.02a
PPOC (mg kg^{-1})	7.79±2.77a	3.48±0.61a	5.48±0.98a	6.25±1.38a	7.12±1.74a	6.59±0.87a	5.30±1.05a	6.64±1.22a	9.41±2.86a
AHOC (g kg^{-1})	2.86±1.33a	6.72±2.76a	3.52±1.76a	2.34±0.64a	5.23±2.82a	6.42±3.71a	9.79±3.55a	10.42±1.22a	10.61±6.14a
AN (mg kg^{-1})	6.69±0.89a	5.21±0.37a	6.68±0.66a	6.69±0.71a	6.92±0.82a	10.55±1.06b	7.51±1.08a	7.51±1.14a	7.76±0.50a
AP (mg kg^{-1})	31.36±11.01a	19.17±1.33a	17.04±0.59a	15.67±1.55a	17.06±0.40ab	20.96±1.34b	14.57±2.77a	19.44±0.65a	19.49±0.81a
MBC (mg kg^{-1})	162.86±31.88ab	142.59±56.31a	340.59±67.81b	109.41±59.78a	79.18±8.20a	207.22±31.69a	64.17±1.93a	141.97±42.06a	270.48±136.64a
MBN (mg kg^{-1})	22.74±4.55a	20.69±4.63a	36.39±10.65a	23.24±2.60a	24.68±0.95a	30.39±5.60a	12.41±3.68a	31.44±1.02b	24.46±7.75ab
MBP (mg kg^{-1})	2.36±1.30a	2.91±1.10a	5.40±0.40a	6.62±3.41a	5.00±0.14a	5.95±1.21a	5.05±1.67a	5.99±0.96a	4.01±1.10a

ST, MT and LT denote short-term, medium-term and long-term treatment respectively. AC, EC1 and EC2 denote the treatments of ambient environment, 550 ppm and 700 ppm CO_2 concentration respectively. Different lowercase letters indicate significant differences ($P < 0.05$) among treatments with different concentrations within the same period. pH, the soil acidity or alkalinity; SOC, soil organic carbon; TN, total soil nitrogen; TP, total phosphorus; DOC, dissolved organic carbon; PPOC, permanganate-oxidizable organic carbon; AHOC, acid hydrolyzed organic; AP, available phosphorus; AN, available nitrogen; MBC, microbial biomass carbon; MBN, microbial biomass nitrogen; MBP, microbial biomass phosphorus.

Table S3 Characteristics of basic environmental factors of soil under the duration of eCO_2 conditions

Soil variables	Treatment					
	EC1-ST	EC1-MT	EC1-LT	EC2-ST	EC2-MT	EC2-LT
pH	5.31±0.03a	5.38±0.05a	5.76±0.12b	5.25±0.04a	5.43±0.10ab	5.62±0.06b
SOC (g kg^{-1})	27.06±0.03a	31.51±1.69a	30.17±1.84a	30.98±3.17a	32.86±3.56a	38.07±2.84a
TN (g kg^{-1})	2.53±0.43a	4.07±0.37a	3.78±0.56a	2.85±0.43a	3.65±0.36a	4.30±1.00a
TP (g kg^{-1})	0.46±0.02a	0.66±0.04a	0.65±0.06a	0.55±0.08a	0.66±0.04a	0.73±0.15a
DOC (g kg^{-1})	0.12±0.01a	0.18±0.02a	0.13±0.02a	0.11±0.001a	0.17±0.03a	0.11±0.02a
EOC (mg kg^{-1})	3.48±0.61a	7.12±1.74a	6.64±1.22a	5.48±0.98a	6.59±0.87a	9.41±2.86a
AHOC (g kg^{-1})	6.72±2.76a	5.23±2.82a	10.42±1.22a	3.52±1.76a	6.42±3.71a	10.61±6.14a
AN (mg kg^{-1})	5.21±0.37a	6.92±0.82a	7.51±1.14a	6.68±0.66a	10.55±1.06b	7.76±0.50a
AP (mg kg^{-1})	19.17±1.33a	17.06±0.40a	19.44±0.65b	17.04±0.59a	20.96±1.34b	19.49±0.81ab
MBC (mg kg^{-1})	142.59±56.31a	79.18±8.20a	141.97±42.06a	340.59±67.81a	207.22±31.69a	270.48±136.64a
MBN (mg kg^{-1})	20.69±4.63ab	24.68±0.95a	31.44±1.02b	36.39±10.65a	30.39±5.60a	24.46±7.75a
MBP (mg kg^{-1})	2.91±1.10a	5.00±0.14a	5.99±0.96a	5.40±0.40a	5.95±1.21a	4.01±1.10a

ST, MT and LT denote short-term, medium-term and long-term treatment respectively. AC, EC1 and EC2 denote the treatments of ambient environment, 550 ppm and 700 ppm CO_2 concentration respectively. Different lowercase letters indicate significant differences ($P < 0.05$) among treatments with different concentrations within the same period. pH, the soil acidity or alkalinity; SOC, soil organic carbon; TN, total soil nitrogen; TP, total phosphorus; DOC, dissolved organic carbon; PPOC, permanganate-oxidizable organic carbon; AHOC, acid hydrolyzed organic; AP, available phosphorus; AN, available nitrogen; MBC, microbial biomass carbon; MBN, microbial biomass nitrogen; MBP, microbial biomass phosphorus.

Table S4 Influencing factors of soil aggregate fractions under eCO_2 and duration of eCO_2

	pH	SOC	TN	TP	DOC	PPOC	AHOC	AN	AP	MBC	MBN	MBP	B	F	F/B	Total PLFAs	Shannon	ACE
ECO_2	0.29*	0.22	0.14	0.15	0.10	0.16	0.22	0.18*	0.12	0.05	0.02	0.14	0.32*	0.47*	0.53*	0.37*	0.18	0.34*
Duration of eCO_2	0.42*	0.03	0.11	0.22	0.05	0.17	0.03	0.09	0.18	0.21	0.32*	0.07	0.36*	0.41*	0.54*	0.36*	0.07	0.31

pH, soil acidity or alkalinity; SOC, soil organic carbon; TN, total soil nitrogen; TP, total phosphorus; DOC, dissolved organic carbon; PPOC, permanganate-oxidizable organic carbon; AHOC, acid-hydrolyzable or-ganic carbon; AP, available phosphorus; AN, available nitrogen; MBC, microbial biomass carbon; MBN, microbial biomass nitrogen; MBP, microbial biomass phosphorus; B, Bacterial phospholipid fatty acid concentration; F, Fungal phospholipid fatty acid concentration; F/B, Fungal to bacterial phospholipid fatty acid concentration ratio; Total PLFAs, total phospholipid fatty acid concentration; Shannon, alpha diversity index; ACE, Microbial richness index.

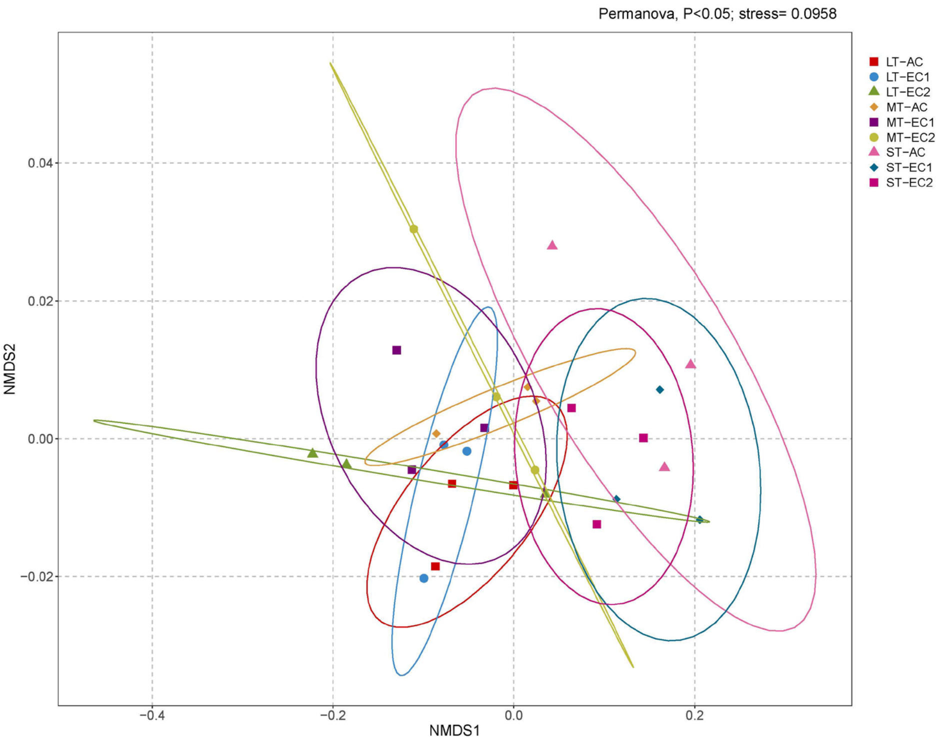

Figure S1. Changes in soil microbial community structure. ST, MT, and LT denote short - term, medium - term,

and long - term treatments respectively; AC, EC1, and EC2 denote treatments under ambient CO_2 concentration, 550 ppm CO_2 concentration, and 700 ppm CO_2 concentration respectively.

Aggregate structure distribution

eCO_2 exposure induced clear changes in soil aggregate size distribution across different exposure durations (Figure S2). Under ST conditions, the aggregate ratio changed from 1:11.5:10:2.5 under ambient CO_2 (AC) to 1:14.7:14.7:3 under EC1 and 1:15:13.3:4 under EC2. For medium-term (MT) aggregates, the ratio shifted from 1:19:16.5:13.5 (AC) to 1:17:18:14.5 (EC1) and 1:40:31:27 (EC2). Under long-term (LT) conditions, the ratio transitioned from 1:8.6:10.7:13 (AC) to 1:36:35:28 (EC1) and 1:18.5:15:15 (EC2) (Figure S2).

Furthermore, the duration of eCO_2 exerted a significant influence on aggregate composition. At EC1 concentration, the aggregate ratios were 1:14.7:14.7:3 (ST), 1:17:18:14.5 (MT), and 1:36:35:28 (LT). Under EC2, the corresponding ratio were 1:15:13.3:4 (ST), 1:40:31:27 (MT), and 1:18.5:15:15 (LT) (Figure S2).

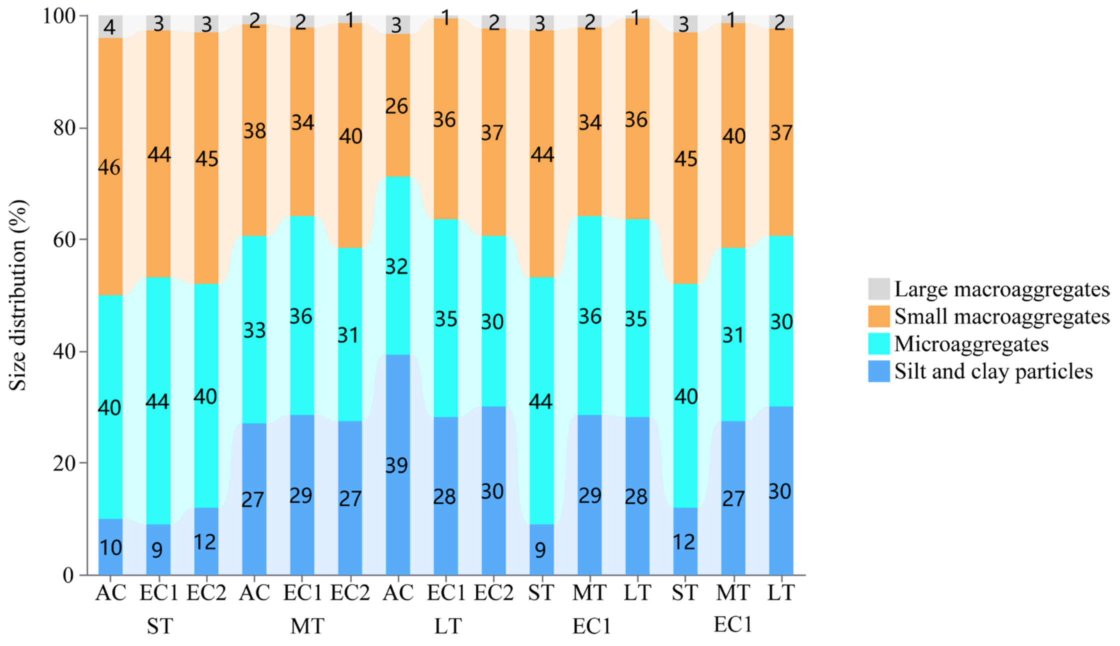

Figure S2. Changes in particle size structure under eCO_2 and the duration of eCO_2. ST, MT, and LT denote short - term, medium - term, and long - term treatments respectively; AC, EC1, and EC2 denote treatments under ambient CO_2 concentration, 550 ppm CO_2 concentration, and 700 ppm CO_2 concentration respectively.
